# miR-25, integrin and cancer invasiveness

**DOI:** 10.18632/oncoscience.214

**Published:** 2015-08-24

**Authors:** Eugenio Zoni, Marianna Kruithof-de Julio, Gabri van der Pluijm

**Affiliations:** Department of Urology, Leiden University Medical Center, Leiden, the Netherlands

**Keywords:** miR-25, microRNA, prostate cancer, integrins, metastasis

Prostate cancer is the most common cancer amongst men worldwide and major cause of cancer death. Current treatments of primary prostate tumors are initially effective, however, 25% of the patients develop metastatic disease. Once the cancer has acquired resistance to Androgen Deprivation Therapy, Castration Resistant Prostate Cancer (CRPC) occurs. At this stage, highly aggressive prostate cancer cells may have spread throughout the body and can form incurable metastases.

Prostate cancer consist of heterogeneous malignant epithelial cell subpopulations of which prostate cancer cells with stem/progenitor-like characteristics have been extensively recognized as the “driver” cancer cell subpopulation in tumor initiation, local and distant relapse, hormone refractory disease, castration, metastasis and chemotherapy resistance [1, 2]. The molecular mechanisms underlying these processes and responsible for the maintenance of the “driver” cancer cell subpopulations have remained largely elusive. MicroRNAs (miRNA, miR) play pivotal roles in the regulation of various cancers, including those of the human prostate [3]. Most studies describe miRNA expression in heterogeneous prostate cancer cell lines or bulk clinical prostate cancer tissues. A major limitation of these approaches is the impossibility to discriminate between stem-like and more differentiated cancer cell subpopulations within these heterogeneous cell populations.

As a result we examined the expression of miRNAs in the subpopulation of highly tumorigenic and migratory stem/progenitor-like tumor cells vs less tumorigenic, sessile cells in both cell lines and patient samples. We found that miR-25 was strongly down-regulated in highly tumorigenic and metastatic stem/progenitor-like prostate cancer cells (ALDH^high^) versus non-tumorigenic, non-metastatic cells (ALDH^low^) [4]. Similarly, in the transformed stem-like epithelial subpopulation isolated from clinical primary prostate cancer tissues (hormone-naïve prostate cancer (with Gleason >7 and CRPC) miR-25 was significantly reduced compared to the transit-amplifying (TA) and committed basal cells (CB)) [5]. In order to understand the biological role of miR-25 in this context, we performed an *in silico* analysis to identify novel miR-25 predicted target genes. Subsequently the genes were clustered into molecular pathways leading to the identification of biological processes linked to invasion, prostate cancer and bone metastasis formation, such as regulation of F-actin cytoskeleton and extracellular matrix-receptor interactions.

Interestingly, αv- and α6-integrin (ITGAV and ITGA6 respectively) were identified among the novel predicted target genes. Both integrins were previously shown to be expressed in prostate epithelial progenitor cells and we previously found that αv-integrins are required for 1) the acquisition/maintenance of a stem/progenitor phenotype in human prostate cancer and 2) acquisition of a migratory and invasive phenotype [6].

Furthermore miR-25 overexpression significantly reduced αv- and α6-integrin mRNAs and protein expression in human prostate cancer cells. Moreover miR-25 also downregulated αv- and α6-integrin protein expression particularly in the selected subpopulation of highly metastatic ALDH^high^ cancer stem/progenitor-like cells. Consistent with these findings, we validated for the first time that αv and α6-integrin mRNAs are direct targets of miR-25 through direct interaction with the 3′ UTR of their respective mRNAs [7].

**Figure 1:**
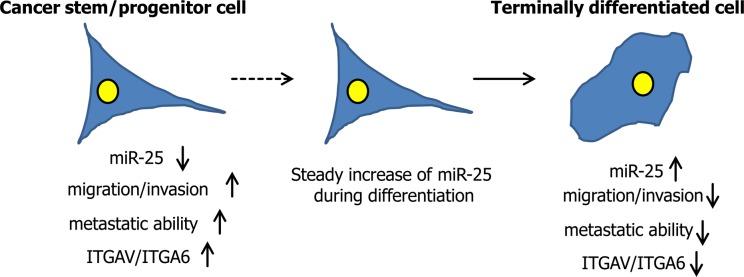
The role of miR-25 in prostate cancer cell invasion and dissemination

In line with the validation of αv- and α6-integrin as direct targets of miR-25, a significant reduction of cellular migration was observed upon enforced expression of miR-25 in the highly motile and metastatic ALDH^high^ subpopulation of cancer stem/progenitor-like cells. When miR-25 overexpressing PC-3M-Pro4 prostate cancer cells were micro-injected into the blood circulation system of embryonic zebrafish embryos, the extravasation and formation of distant metastasis was strongly impaired versus control cells expressing a scramble sequence.

Taken together our data show that miR-25 negatively regulates the acquisition of an invasive and metastatic phenotype in human prostate cancer cells. Mir-25 steadily increases during epithelial differentiation in both the non-transformed and transformed human prostate (stem-cell->transit amplifying cell-> committed basal cells). This inverse relationship between miR-25 expression and differentiation of transformed prostate epithelial cells underscores the key regulatory role of miR-25 in the regulation of prostate cancer malignancy. We showed that through the modulation an invasive, stem-like, tumorigenic phenotype, miR-25 seems to play a role in the differentiation into a more epithelial, sessile, less tumorigenic subpopulation of human prostate cancer cells. Our findings also support the notion that interpretation of mRNA or miR profiles of bulk clinical tissues needs to be carried carefully because (prostate) cancer tissues are notoriously heterogeneous.
